# Quantitative and Qualitative Analysis of Aconitum Alkaloids in Raw and Processed Chuanwu and Caowu by HPLC in Combination with Automated Analytical System and ESI/MS/MS

**DOI:** 10.1155/2012/936131

**Published:** 2012-04-11

**Authors:** Aimin Sun, Bo Gao, Xueqing Ding, Chi-Ming Huang, Paul Pui-Hay But

**Affiliations:** ^1^Analytical & Testing Center, Sichuan University, Chengdu 610064, China; ^2^Government Laboratory of Hong Kong, Kowloon, Hong Kong; ^3^Department of Biology, Chinese University of Hong Kong, Shatin, N.T., Hong Kong

## Abstract

HPLC in combination with automated analytical system and ESI/MS/MS was used to analyze aconitine (A), mesaconitine (MA), hypaconitine (HA), and their benzoyl analogs in the Chinese herbs Caowu and Chuanwu. First, an HPLC method was developed and validated to determine A, MA, and HA in raw and processed Caowu and Chuanwu. Then an automated analytical system and ESI/MS/MS were applied to analyze these alkaloids and their semihydrolyzed products. The results obtained from automated analytical system are identical to those from ESI/MS/MS, which indicated that the method is a convenient and rapid tool for the qualitative analysis of herbal preparations. Furthermore, HA was little hydrolyzed by heating processes and thus it might account more for the toxicity of processed aconites. Hence, HA could be used as an indicator when one alkaloid is required as a reference to monitor the quality of raw and processed Chuanwu and Caowu. In addition, the raw and processed Chuanwu and Caowu can be distinguished by monitoring the ratio of A and MA to HA.

## 1. Introduction

Aconite rootstocks of *Aconitum carmichaelii* (Chuanwu) and *A*. *kusnezoffii *(Caowu) have been used in traditional Chinese medicine for over two millennia. They are commonly used as a key ingredient in many herbal prescriptions in the Orient for rheumatalgia, heart failure, contracture of limbs, and pain in joints. The principal active ingredients in Chuanwu and Caowu are alkaloids with C19-diterpenoid skeleton, including aconitine (A), mesaconitine (MA), and hypaconitine (HA) [1, 2]. However, these alkaloids are also toxic and have a very narrow safety range, because they could easily induce ventricular tachycardia and fibrillation even at therapeutic dose levels. Down the ages, various processing methods, including prolonged steaming and boiling, were developed to reduce their toxicity [2, 3]. During the course of steaming and boiling, aconitines would be hydrolyzed to benzoylaconitines and aconitines, which still retain analgesic properties while their toxicity is reduced about a hundred-fold [4]. From 1989 to 2006, over 45 aconite poisoning cases were reported in Hong Kong, among which three cases were fatal [4–6]. Aconite poisoning cases often occur in other Asian countries, as well as, for example, India [7] and Japan [8].

In order to ensure the safety and effectiveness of clinical diagnosis as well as forensic investigation of aconite poisoning, it is necessary to develop convenient, selective, and accurate methods to identify and determine these alkaloids in herbal medicine. Various methods have been published, including IR [9], HPLC [10, 11], HPCE [12], HPLC/MS [13–15], and GC/MS [16]. However, there have been few reports about the analysis of aconite alkaloids in herbal medicine using HPLC in combination with an automated analytical system and ESI/MS/MS. In the present study, an HPLC method was established and validated to determine A, MA, and HA in raw and processed Caowu and Chuanwu. Then an automated analytical system and ESI/MS/MS were applied to the qualitative analysis of these alkaloids and their semihydrolyzed products. The results indicated that HA was the most stable compound among A, MA, and HA, after undergoing the prolonged heating treatment, which suggested that HA plays a major role in the toxicity of aconite alkaloids in processed Chuanwu and Caowu. Hence, HA could be used as an indicator when one alkaloid is required as a reference to monitor the quality of aconite alkaloids. In addition, raw and processed Chuanwu and Caowu can be distinguished by monitoring the ratio of A and MA to HA. Similar situation has been found in Fuzi (young tubers of *Aconitum carmichaeli*) [17].

## 2. Experimental

### 2.1. Chemicals and Materials

The reference standard of A was purchased from Sigma Chemical Co. (St. Louis, U.S.A). The reference standards of MA and HA were purchased from the Institute for the Control of Pharmaceutical and Biological Products, China. Raw and processed herbs (Chuanwu, Caowu) were purchased from herb market in Hong Kong. Benzoylaconitine, benzoylmesaconitine, and benzoylhypaconitine were prepared from A, MA, and HA, respectively, in our laboratory.

LC-grade acetonitrile (ACN) and tetrahydrofuran and analytical-grade phosphoric acid and triethylamine (TEA) were purchased from Riedel-de Haen Co. (Germany). All the other reagents were of analytical grade. Double distilled water was used throughout the study.

### 2.2. Preparation of Standard and Sample Solutions

Three standard solutions were prepared by accurately dissolving the A, MA, and HA in ACN-TEA (75 : 25, v/v) buffer, respectively. Raw and processed herbs (Chuanwu and Caowu) were pulverized into powder. After passing through a 0.45 mm sieve, the powder was dried in an oven at 55°C for 6-7 h, then three samples with 1.0 g weight were accurately weighed. Each weighed sample was extracted with 1 mL aqueous ammonia solution (30%) for 20 min at room temperature and then was extracted with ethyl ether (20 mL) in an ultrasonic bath for 10 min. After the sample was placed at room temperature for 16 h, the liquid phase was filtered. The residue was further extracted for 3 additional times in the same manner (and the final extraction was assured complete). The filtrate was pooled together and was extracted with 2% hydrochloric acid for 4 times (25 mL each time). The aqueous solution was adjusted with ammonia solution to pH 10 and further extracted with ethyl ether for 3 times (25 mL, each time). After being washed with water (10 mL), the combined ether solution was dried with anhydrous sodium sulphate and then was evaporated to dryness at 40°C in an evaporator. The residue was further dissolved with 1 mL ACN-TEA (75 : 25, v/v). All final solutions were filtered through a 0.45 *μ*m filter membrane, and 20 *μ*L filtrate was injected into the HPLC system for analysis. 

## 3. Apparatus

### 3.1. HPLC-DAD Chromatographic System

The Agilent/HP 1090 series HPLC system (Hewlett Packard, CA, USA) consisted of quaternary pump, guard column (Econosphere C18, 5 *μ*m, Alltech, USA), analysis column (25 cm × 4.6 mm i.d., Microsorb C18, 5 *μ*m, USA), and a diode-array detector. The optimal HPLC conditions were as follows: a mixture of ACN-TEA (75 : 25, v/v) was selected as the solvent of standards and samples, while a mixture of ACN, TEA buffer (pH 3.0; 25 mM), and THF was used as the mobile phase in the gradient program, and the flow rate was maintained at 1 mL/min. Detection wavelength was set at 238 nm and the column temperature was maintained at 45°C.

### 3.2. Automated Analytical System

The automated analytical system mainly included an autosampler, four tandem columns (concentration column, extraction column, separation column I, and separation column II), a UV detector, the intelligence software, and a spectral library. Automated analytical system has several distinct advantages, including automated pretreatment and sample analyses, short analysis time, and high specificity. The system was used for the direct qualitative analysis of A, HA, and MA, and sample pretreatment was not necessary in this system.

### 3.3. ESI/MS/MS System

The ESI/MS/MS system consisted of an ESI source and a model MAT TSQ 700 tandem mass spectrometer (Finnigan, CA, USA). The pressure and temperature were set at 15 psi and 145°C, respectively. The ESI source was operated in the positive ion mode, and the high voltage for the cylindrical electrode was set at −4510 V.

In the above experiments, extracted sample dissolved in methanol-water-acetic acid (50 : 50 : 0.005, v/v/v) was directly infused into the ESI source at a rate of 5 *μ*L/min. The mass spectrometer was scanned from 100 to 700 amu per second.

## 4. Results and Discussion

### 4.1. Optimization of Chromatographic Condition

**Figure 1:**
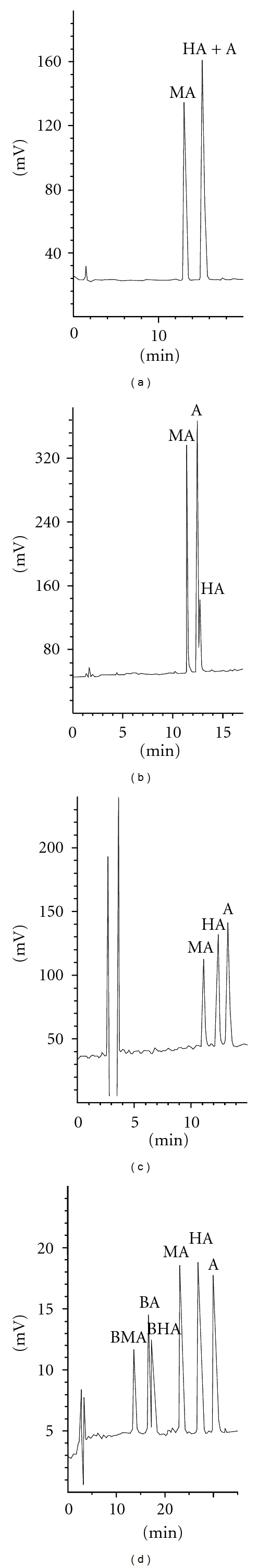
HPLC chromatograms of aconitine (A), mesaconitine (MA), hypaconitine (HA), and their corresponding semi-hydrolyzed products benzoylaconitine (BA), benzoylmesaconitine (BMA), and benzoylhypaconitine (BHA) in different gradient programs (a)–(d) (see Table 1).

Several factors influencing separation of alkaloids were investigated. It was found that the resolutions of aconitine, mesaconitine, and hypaconitine were markedly affected by the concentration of triethylamine phosphate in buffer solution. Since the best separation resolution for the alkaloids was achieved at the concentration of less than 30 mM and the background signal noise increased at less than 10 mM, therefore, 25 mM buffer solution was finally chosen as the HPLC mobile phase for subsequent analysis. Gradient program was optimized in detail. Different gradient modes of mobile phases were chosen according to the following gradient programs (Table 1) and the subsequent chromatograms were shown in Figure 1.

The best result in terms of peak shape, resolution, and run time was obtained with a mixture of ACN, TEA buffer (pH 3.0; 25 mM), and THF, in a gradient mode of mode d of Table 1. So the optimal HPLC conditions were as follows: a mixture of ACN-TEA (75 : 25, v/v) was selected as the solvent of standard and samples, and a mixture of ACN-TEA buffer (pH 3.0; 25 mM) and THF was used as the mobile phase in a gradient mode of 0 : 90 : 10 (0 min), 6 : 84 : 10 (20 min), and 26 : 64 : 10 (40 min).

### 4.2. Validation of HPLC Assay

The regression equation, linear range, correlation coefficient (*r*), and limit of detection obtained from the established HPLC method for the assay of A, MA, and HA are listed in Table 2. The intraday relative standard deviations (3 run for each concentration) were less than 1.61%, and interday relative standard deviations (3 run per day for each concentration within 3 consecutive days) were less than 7.3%. The average recovery of A, MA, and HA was found to be 91%, 89%, and 87%, respectively.

### 4.3. Quantitative Determination of A, HA, and MA in Raw and Processed Aconites

**Figure 2:**
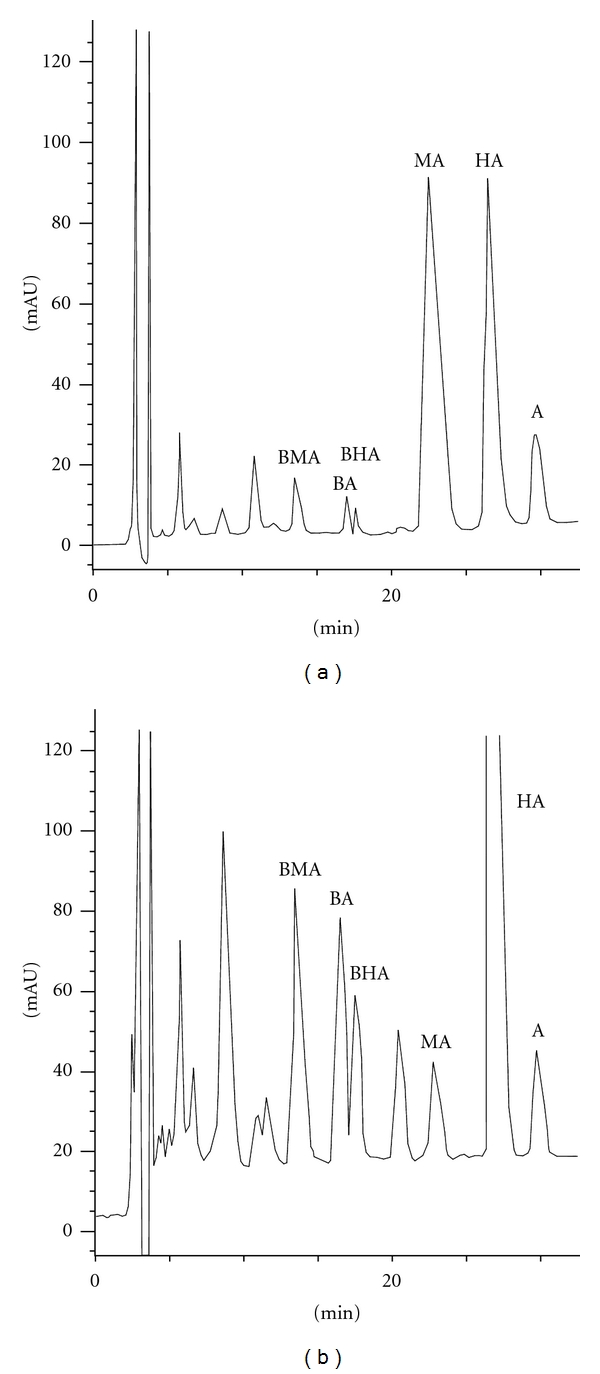
HPLC chromatograms of raw Chuanwu (a) and processed Chuanwu (b).

A, MA, and HA in raw and processed aconite samples were determined by the established HPLC method. The analytical results are shown in Table 3. It could be seen that the contents of the three alkaloids in processed Chuanwu samples were much lower than those in raw Chuanwu samples (Figures 1(d) and 2), the apparent reason of which was that these aconite alkaloids in raw Chuanwu were hydrolyzed to their corresponding semi-hydrolyzed products during the treating process.

In addition, we have also conducted preliminary study to identify the benzoylaconitines hydrolysed from the three alkaloids in raw and processed Chuanwu and Caowu, by heating A, MA, and HA in dioxin/H_2_O (7.5/2.5, v/v) at 120°C for 50 min. Benzoylaconitine, benzoylmesaconitine and benzoylhypaconitine were obtained from A, MA, and HA, which lost the acetal groups during the hydrolysis process. After heating treatment, most of A and MA transformed to benzoylaconitine analogues, while, HA had little change, which makes its content the highest among the three alkaloids (A, MA, and HA) in the processed herbs. Moreover, the results, obtained from boiling raw herbs in water for 15, 30, 60, 90 and 150 min, revealed that contents of A and MA dropped rapidly with an increase in boiling time, and A and MA disappeared completely at 150 min. However, the reduction of HA was small after the same treatment, and its amount still remained significant even at 150 min. It is interesting that HA could survive the heating process, which suggested that HA might play a major role in the toxicity of aconitum alkaloids in processed Chuanwu and Caowu. Hence, it could be used as an indicator when one alkaloid is required as a reference to monitor the quality of aconite alkaloids. In addition, the raw and processed Chuanwu and Caowu can be distinguished by monitoring the ratio of A and MA to HA.

### 4.4. Qualitative Analysis of A, MA, and HA by Automated Analytical System

**Figure 3:**
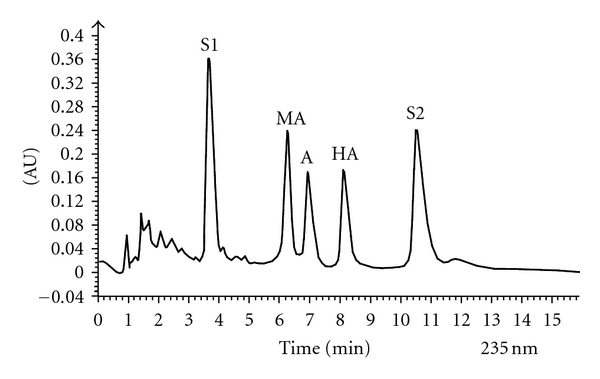
A chromatogram of A, MA, and HA obtained by automated analytical system (S1, S2 are internal standards).

In the qualitative analysis of these alkaloids, the standard solution of A was injected into the automated analytical system. The spectral fingerprint parameters of the unknown sample are shown in Table 4 and are compared with those of the candidates suggested by the intelligence software. The searching results for match-fingerprint parameters in the spectral library proved that the unknown sample was A. The identification of MA and HA was achieved by the same procedure. The automated analytical system chromatogram of a mixed standard (A, MA, HA) is shown in Figure 3.

### 4.5. Qualitative Analysis of A, MA, HA, and Their Hydrolyzed Analogs by ESI/MS/MS

**Figure 4:**
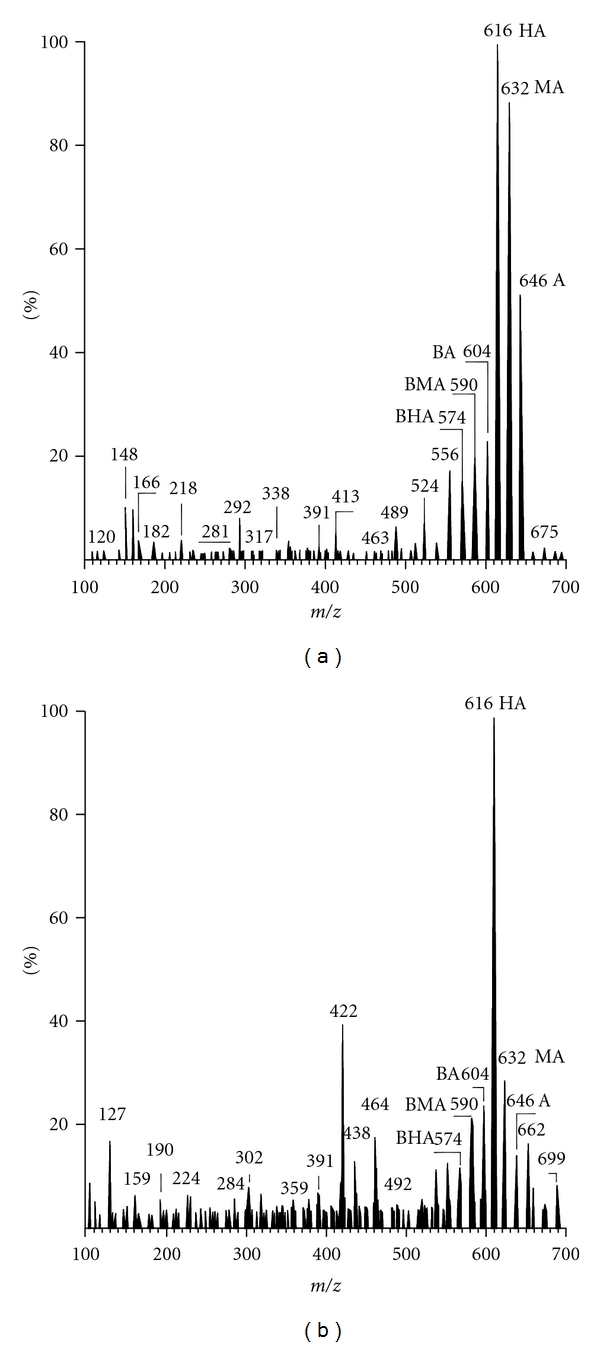
The ESI/MS spectra of (a) the mixture of A, MA, and HA standards at 1 *μ*g/mL level; (b) the decocted Chuanwu (the time of decoction was 0.5 h).

To confirm the results obtained by automated analytical system, aconite alkaloids were then analyzed by ESI/MS/MS. A, MA, and HA are C19-diterpenoid alkaloids containing a nitrogen atom. The mass spectra showed the fragments of protonated molecules ([M+H]^+^) and the characteristic losses of side chain CH_3_COOH (60), C_6_H_5_COOH (122), *m*/*z* 152, and 279 (Table 5). The ESI/MS spectra of a mixture of A, MA, and HA standards at 1 *μ*g/mL level and a decocted Chuanwu (the time of decoction was 0.5 h) were shown in Figure 4. The mass spectra showed [M+H]^+^ ion at *m*/*z* 646 for A, at *m*/*z* 632 for MA, at *m*/*z* 616 for HA, at *m*/*z* 603 for benzoyl-aconitine, at *m*/*z* 589 for benzoyl-mesaconitine, and at *m*/*z* 573 for benzoyl-hypaconitine. The protonated molecular ion has minimal fragmentation because of the soft ionization of the ESI.

As shown in Figure 4(a), the [M+H]^+^ peaks of the aconite alkaloids were the characteristic peaks, which could be used to simultaneously identify these alkaloids, though the sample was directly introduced into the ESI/MS.  Figure 4(b) shows the relative amounts of the three aconite alkaloids and semi-hydrolyzed products in a decocted Chuanwu. The results indicated that HA was the major component in Chuanwu decoctions. The identity of each alkaloid could be further confirmed by ESI/MS/MS. After undergoing CID (collision-induced decomposition) by argon gas, the protonated molecular ion (the parent ion, selected by the first mass analyzer) was transformed into fragments, which could facilitate structural identification of the parent ion. 

**Figure 5:**
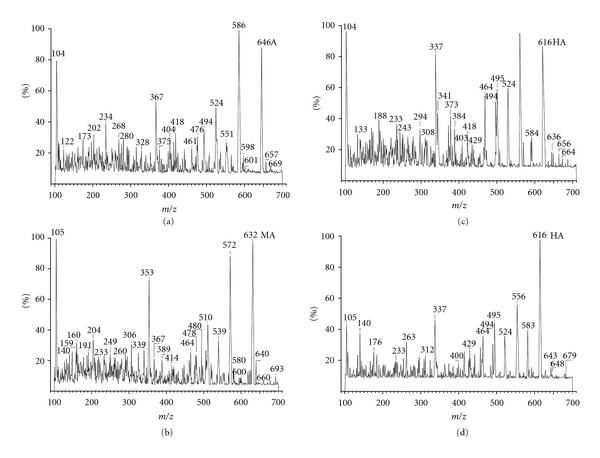
ESI/MS/MS spectra of A standard (a), HA standard (c), MA standard (b), and HA in decocted Chuanwu (d).

It can be seen from Figures 5(a), 5(b), and 5(c) that the protonated molecule ions ([M+H]^+^) of A, MA, HA are at *m*/*z* 646, 632, and 616, respectively, and the major fragment ions (the neutral loss 60, 122, 152, 279) are at *m*/*z* 524, 494, 367 for A, *m*/*z* 510, 480, 353 for MA, and *m*/*z* 494, 464, 337 for HA, respectively. Figure 5(d) shows that the ESI/MS/MS spectrum of HA in decocted Chuanwu is the same as that of the standard HA.

## 5. Conclusions

HPLC in combination with automated analytical system and ESI/MS/MS was employed to analyze A, MA, HA, and their benzoyl analogs in our study. The analytical results obtained from of automated analytical system are identical to those from ESI/MS/MS. Therefore, the automated analytical system would be a good complementary method for the quality control of the herbal preparations containing aconite alkaloids. The most important finding in this research is that the analytical results indicated that HA was the most stable compound among A, MA, and HA, even after prolonged heating treatment, and thus suggest that HA might play a major role in the toxicity of processed Chuanwu and Caowu. Hence, HA could be used as an indicator when one alkaloid is required as a reference to monitor the quality of aconitum alkaloids, and the raw and processed Chuanwu and Caowu can be distinguished by monitoring the ratio of A and MA to HA.

## Figures and Tables

**Table 1:** Different gradient elution programs for the HPLC analysis of A, MA, HA, and their correlative semi-hydrolyzed products.

No.	Concentration	0 min	20 min	40 min
Mode a	ACN	10	30	50
	TEA buffer	90	70	50
Mode b	ACN	0	90	90
	TEA buffer	100	10	10
Mode c	ACN	0	80	80
	TEA buffer	90	10	10
	THF	10	10	10
Mode d	ACN	0	6	26
	TEA buffer	90	84	64
	THF	10	10	10

**Table 2:** The regression equation, linear range, correlation coefficient (*r*), and limit of detection obtained from the established HPLC method for the assay of aconitine (A), mesaconitine (MA), and hypaconitine (HA).

Alkaloids	Linear range (*μ*g/mL)	Regression equation	Correlation coefficient (*r*)	Detection limit (ng)
Aconitine	2.50–505	*Y* = 2.270X + 9.490	0.9999	49
Mesaconitine	2.45–490	*Y* = 1.917X + 4.010	0.9998	51
Hypaconitine	2.50–500	*Y* = 11.405X + 35.131	0.9999	50

**Table 3:** HPLC analytical of raw and processed Caowu and Chuanwu samples.

Sample		Found (*μ*g/g)*	
	Aconitine	Mesaconitine	Hypaconitine
Raw Caowu	83.7 ± 6.9	527.1 ± 66.7	240.5 ± 6.4
Processed Caowu	4.5 ± 2.1	4.7 ± 0.8	24.0 ± 1.4
Raw Chuanwu	117.5 ± 5.0	421.5 ± 48.7	318 ± 41.0
Processed Chuanwu	12.6 ± 4.2	23.8 ± 4.9	38.1 ± 2.9

*Average ± standard deviation (*n* = 3).

**Table 4:** The spectral fingerprint parameters of the unknown sample obtained by automated analytical system.

Parameter	Data from	Data from
	unknown sample	spectral library
Wavelength_max_	242	240
RRT1	2.330	2.418
RRT2	0.531	0.515
Ratio 1	0.391	0.400
Ratio 2	0.850	0.860
Ratio 3	0.991	1.030
2 DI	251	249
SF	0.111	

**Table 5:** Protonated molecular ions and characteristic fragment ions of aconitum alkaloids obtained by ESI/MS/MS.

Compound	[M+H]^+^ (*m*/*z*)	Major fragment ion (*m*/*z*)	(Neutral loss 60 122 152 279)		
Aconitine	646	586 [M+H–CH_3_COOH]^+^	524	494	367
Mesaconitine	632	572 [M+H–CH_3_COOH]^+^	510	480	353
Hypaconitine	616	556 [M+H–CH_3_COOH]^+^	494	464	337
